# Bifidobacteria strains isolated from stools of iron deficient infants can efficiently sequester iron

**DOI:** 10.1186/s12866-014-0334-z

**Published:** 2015-01-16

**Authors:** Pamela Vazquez-Gutierrez, Christophe Lacroix, Tanja Jaeggi, Christophe Zeder, Michael Bruce Zimmerman, Christophe Chassard

**Affiliations:** Laboratory of Food Biotechnology, ETH Zurich, Institute of Food, Nutrition and Health, Schmelzbergstrasse 7, Zurich, Switzerland; Laboratory of Human Nutrition, ETH Zurich, Institute of Food, Nutrition and Health, Schmelzbergstrasse 7, Zurich, Switzerland

**Keywords:** Bifidobacteria, Iron, Siderophore, Nutrient competition, CAS assay

## Abstract

**Background:**

Bifidobacteria is one of the major gut commensal groups found in infants. Their colonization is commonly associated with beneficial effects to the host through mechanisms like niche occupation and nutrient competition against pathogenic bacteria. Iron is an essential element necessary for most microorganisms, including bifidobacteria and efficient competition for this micronutrient is linked to proliferation and persistence. For this research we hypothesized that bifidobacteria in the gut of iron deficient infants can efficiently sequester iron. The aim of the present study was to isolate bifidobacteria in fecal samples of iron deficient Kenyan infants and to characterize siderophore production and iron internalization capacity.

**Results:**

Fifty-six bifidobacterial strains were isolated by streaking twenty-eight stool samples from Kenyan infants, in enrichment media. To target strains with high iron sequestration mechanisms, a strong iron chelator 2,2-dipyridyl was supplemented to the agar media. Bifidobacterial isolates were first identified to species level by 16S rRNA sequencing, yielding *B. bifidum* (19 isolates), *B. longum* (15), *B. breve* (11), *B. kashiwanohense* (7), *B. pseudolongum* (3) and *B. pseudocatenulatum* (1)*.* While most isolated bifidobacterial species are commonly encountered in the infantile gut, *B. kashiwanohense* was not frequently reported in infant feces. Thirty strains from culture collections and 56 isolates were characterized for their siderophore production, tested by the CAS assay. Siderophore activity ranged from 3 to 89% siderophore units, with 35 strains (41%) exhibiting high siderophore activity, and 31 (36%) and 20 (23%) showing intermediate or low activity. The amount of internalized iron of 60 bifidobacteria strains selected for their siderophore activity, was in a broad range from 8 to118 μM Fe. Four strains, *B. pseudolongum* PV8-2, *B. kashiwanohense* PV20-2, *B. bifidum* PV28-2a and *B. longum* PV5-1 isolated from infant stool samples were selected for both high siderophore activity and iron internalization.

**Conclusions:**

A broad diversity of bifidobacteria were isolated in infant stools using iron limited conditions, with some strains exhibiting high iron sequestration properties. The ability of bifidobacteria to efficiently utilize iron sequestration mechanism such as siderophore production and iron internalization may confer an ecological advantage and be the basis for enhanced competition against enteropathogens.

**Electronic supplementary material:**

The online version of this article (doi:10.1186/s12866-014-0334-z) contains supplementary material, which is available to authorized users.

## Background

Bifidobacteria represent one of the most predominant groups of commensal bacteria residing in the human and animal gastrointestinal tract [1]. *Bifidobacterium* spp. are among the first anaerobes able to reach high levels within the first week of life and generally represent more than 40% of infant gut microbiota [2,3]. Different *Bifidobacterium* species are associated with a broad range of beneficial effects on host’s health. Their colonization in the gut is associated with modulation of the intestinal microbiota composition and activity, immune-modulation and attenuation of inflammatory symptoms [4,5]. Additionally, mechanisms involved in host protection include nutrient competition, competitive exclusion, direct antagonism by inhibitory substances and host-mediated effects such as improved barrier function and altered immune response [6,7]. Numerous studies have demonstrated the efficacy against enteric pathogens of selected bifidobacterial strains but the mechanistic basis supporting the observed benefits is still often lacking [8]. Strain dependency is one of the features that have been revealed while seeking for new probiotic strains [9], thus the importance of selection of suitable candidates through biochemical assays targeting potential probiotic functions.

Iron is an essential micronutrient for most intestinal commensal and pathogenic bacteria, except for *Lactobacillus* [10]. Iron is involved in many essential metabolic processes like cell proliferation, electron transport, and is a cofactor for different enzymes [11]. For instance, iron plays an important role in most pathogenic bacteria, as iron sequestration mechanism have been associated to cell replication and persistence and thus being involved in pathogenesis [12]. However, iron is restricted in most environments including the mammalian intestine [13]. The limited availability of iron in the host provides one form of non-specific immune-defence that bacteria, for instance, bacterial pathogens need to overcome in order to grow and cause infection [14]. Therefore microorganisms have evolved different acquisition systems like import of iron from different sources and production of siderophores to utilize, accumulate and sequester iron [15]. Various physiological mechanisms such as low/high affinity iron transport systems are employed by bacteria to tightly control iron uptake, intracellular concentration and storage, preventing toxic effects [16]. Iron starvation signals the up-regulation of acquisition systems and as a response many gram positive bacteria synthesize low molecular weight iron chelating ligands, known as siderophores, to compete for iron [17]. In contrast, iron excess activates the expression of efflux pumps and ferritin like proteins involved in iron accumulation [18,19].

Withholding iron has been identified as a major competitive and defense mechanisms in many gram positive and gram negative bacteria, because of its general limited availability in different environments, such as the intestine [20,21]. The concept of restricting iron to pathogens has been coined as nutritional immunity [22,23] and is usually associated with efficient iron sequestration systems. It has been hypothesized that one of the beneficial actions of health promoting bifidobacteria is to sequester iron, thus making it less available to pathogens, hence representing another form of nutritional immunity [24]. The ability of bifidobacteria to outcompete by producing siderophores and/or concentrating iron from the environment may therefore provide a competitive advantage against competing microorganism by limiting the iron available in complex ecosystems such as the gut microbiota. Thus, bifidobacteria, may cause iron starvation of competing microorganism; for instance, enteropathogens. However to our knowledge, the ability to produce siderophores in culture supernatant and to internalize iron of diverse bifidobacteria species has never been investigated. We hypothesized that bifidobacteria present in the gut of iron deficient infants efficiently sequester iron. Therefore our aim was to isolate *Bifidobacterium* strains from stool samples from iron deficient Kenyan infants and to investigate their ability to produce siderophores as well as to sequester iron in comparison to culture collection strains.

## Results

### Isolation and genotypic identification

The bifidobacteria gene copy numbers (CN) was, assessed in the twenty-eight stool samples of Kenyan infants by qPCR targeting the xylulose-5-phosphate/fructose-6-phosphatephosphoketolase gene (*xfp)* gene. The mean bifidobacteria CN was log 9.8 SD +/− 0.4 copies per gram feces, in the range from log 6.4 to log 10.4 copies per gram feces sample. Twenty-eight baseline stool samples from Kenyan infants were streaked in BRS, MRS-cys, TPY and BSM agar supplemented with a strong iron chelator 2,2-dipiridyl. A total of 208 strains were isolated and analyzed by 16S rRNA gene sequencing for identification. 16S rRNA partial sequences with a length of 1100 to 1300 bp after quality trimming were aligned for genotypic identification using BLAST, yielding 41% *Enterococcus* spp. (94 isolates), 29% *Bifidobacterium* spp. (56), 28% *Lactobacillus* spp. (54), 1% *Weisella* spp. (2), 0.5% *Streptoccocus* spp. (1) and 0.5% *Leuconostoc* spp. (1). *Bifidobacterium* 16S rRNA partial sequences were assigned to six phylogenetic taxa: 34% *B. bifidum* (19 isolates), 27% *B. longum* (15)*,* 19% *B. breve* (11), 13% *B. kashiwanohense* (7), 5% *B. pseudolongum* (3), 2% *B. pseudocatenulatum* (1) (Table 1). *B. bifidum* was most frequently isolated in BSM and Beerens agar, while *B. longum* and *B. breve* were obtained from TPY plates and *B. kashiwanohense* from TPY and BSM plates. *Bifidobacterium* isolated on BSM agar represented five species (5), compared to four species isolated on Beerens, three on TPY and only one on MRS-cys (1) agar. Three different bifidobacteria species were isolated from each stool samples 3, 5, 8 and 11 (Table 2), while either one or two bifidobacterial species were obtained from the other samples. In addition, *B. kashiwanohense* was only found in three fecal samples 11, 20 and 25 (Table 2).

**Table 1:** **Occurrence and isolate numbers of isolated** ***Bifidobacterium*** **species in the infant stool samples (n = 28)**

	**Distribution of bifidobacterial species in infant fecal samples**	**No. isolates obtained**
	**n = 28**	**n = 56**
*B. bifidum*	13	19
*B. longum*	8	15
*B. breve*	8	11
*B. kashiwanohense*	3	7
*B. pseudolongum*	1	3
*B. pseudocatenulatum*	1	1

**Table 2:** **Bifidobacterial population and species isolated from Kenyan infant stool samples (n = 28)**

**Feces sample**	**Hb (g/dl)**	**Total bacteria**	**Bifidobacteria population**	**Isolated species from different iron restricted media**
1	10.1	11.6	9.7	*B. breve* BSM1-2
2	8.7	10.4	9.4	*B. bifidum* BSM2-1, *B. bifidum* BSM2-3*, B. breve* TPY2-1, *B. breve* TPY2-3, *B. breve* TPY2-4
3	10.2	10.9	9.5	*B. breve* BSM3-1, *B. longum* TPY3-1*, B. longum* TPY3-2, *B. pseudocatenulatum* BRS3-2*, B. bifidum BRS-300*
4	9.9	11.8	6.4	*B. longum* TPY4-1
5	9.6	11.3	8.9	*B. longum* PV5-1, *B. bifidum* BRS5-3*, B. breve* TPY5-1, *B. breve* TPY5-2
6	9.2	10.3	9.0	*B. bifidum* TPY6-2, *B. bifidum* MRSc6-292, *B. bifidum* MRSc6-312
7	8.6	10.8	9.5	No *Bifidobacterium* isolates obtained
8	8.1	11.7	10.0	*B. pseudolongum* PV8-2, *B. pseudolongum* BSM8-1, *B. pseudolongum* BSM8-3, *B. breve* BSM8-4, *B. bifidum* BRS8-1*, B. bifidum* BRS8-2, *B. bifidum* BRS8-3, *B. longum* TPY8-1, *B. longum* TPY8-2
9	9.9	11.1	9.5	No *Bifidobacterium* isolates obtained
10	9.8	11.2	9.9	*B. breve* TPY10-1, *B. breve* TPY10-2
11	9.5	12.1	10.1	*B. kashiwanohense* BSM11-1, *B. kashiwanohense* BSM11-4, *B. longum* BSM11-5, *B. kashiwanohense* TPY11-1, *B. kashiwanohense* BRS11-1, *B. kashiwanohense* TPY11-2
12	11.3	12.2	9.7	*B. bifidum* BSM12-2*, B. longum* TPY12-1
13	10.3	9.9	9.2	*B. longum* TPY13, *B. longum* TPY13-2, *B. longum* TPY13-3
14	10.4	10.6	9.4	No bifidobacteria isolate obtained
15	10.4	10.4	9.1	*B. bifidum* BSMd15, *B. bifidum* BSM15-2,
16	10.5	10.6	9.3	*B. bifidum* BRS16-1*, B. breve* TPY16-2
17	10.5	10.7	9.4	No *Bifidobacterium* isolates obtained
18	10.8	11.1	9.1	*B. bifidum* BSM18
19	10.8	10.5	8.9	No *Bifidobacterium* isolates obtained
20	11.1	10.7	9.0	*B. kashiwanohense* PV20-2
21	11.3	10.7	9.0	*B. bifidum* BRS21
22	11.4	10.1	8.9	No *Bifidobacterium* isolates obtained
23	11.8	11.7	10.4	No *Bifidobacterium* isolates obtained
24	11.8	11.7	10.2	No *Bifidobacterium* isolates obtained
25	NDA	10.4	9.3	*B. kashiwanohense* TPY25-1
26	NDA	11.1	9.0	*B. bifidum* BRS26-2 *, B. longum* TPY26-1
27	NDA	10.1	9.0	*B. breve* TPY27, *B. bifidum* BRS27-3
28	NDA	12.0	9.7	*B. bifidum* PV28-2a *, B. bifidum* BSM28-1, *B. bifidum* BSMd28-2

Bifidobacterial population is expressed as bifidobacterial log copies per gram feces. SD for bifidobacteria population among samples was +/− 0.35 and for total bacteria SD +/− 0.56. NDA: No data available. Hb: Hemoglobin level (g/dL).

### Characterization based on siderophore production

**Figure 1:**
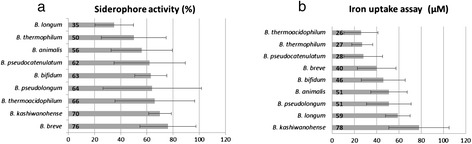
**Siderophore activity (a) and iron internalization within bifidobacterial species (b)** ***.***

Eighty-six bifidobacterial strains comprising 56 isolates and 30 culture collection strains were characterized for their ability to produce siderophores using the CAS assay (Additional file 1: Figure S1). Siderophore activity (SA) was found to be widely distributed and strain dependent among bifidobacterial strains. Percentage of SA ranged from 3% to 89% siderophore units. To group strains according to SA values, the following ranges were established according to OD_630nm_ measurements and color change after mixing with the CAS dye. Three groups were defined: strains with high SA in the range from 90-60% siderophore units and a color change to orange; strains with intermediate SA in the range from 59-30% and a color change to pink, and strains with intermediate SA in the range from 29-0% and no visible color change. Thirty-four strains (41%) belonged to the first group of high SA, 32 strains (36%) showed intermediate SA, and 20 strains (23%) showed low SA. No correlation was found between SA and hemoglobin levels of the infant stool donor where the strains were isolated (Additional file 2: Table S1). From the thirty-four strains showing high SA, 10 strains belonged to public culture collections and 24 strains belonged to the isolates. Compare to *B. bifidum* MRSc6-292, the strain with the lowest SA (3 +/−1%), eight *B. breve*, five *B. bifidum*, three *B. pseudolongum*, four *B. kashiwanohense* and three *B. thermoacidophilum* strains showed significantly higher SA (p < 0.0001). *B. breve* showed the highest SA value among species, with 76 +/− 21% (n = 12) (Figure 1a).

Strains showing similar genotypic identification in the 16S rRNA sequence, isolated from same stool sample and plate and that also showed similar SA value; as well as, strains showing low siderophore activity, were excluded from the iron internalization (II) characterization. Therefore 43 isolates and 19 culture collection strains were available for II characterization.

### DNA finger printing

Phylogenetic diversity of sixty-two *Bifidobacterium* strains included in the II characterization was evaluated by DNA-finger printing using RAPD and REP-PCR (Additional file 3: Figure S2). DNA finger prints showed that strains retained for the II characterization were phylogenetically different, except for *B. breve* TPY 10–2 and *B. pseudolongum* BSM 8–1 as they showed identical DNA finger prints with *B. breve* TPY 10–1 and *B. pseudolongum* BSM 8–2, respectively.

### Characterization based on iron internalization

**Figure 2:**
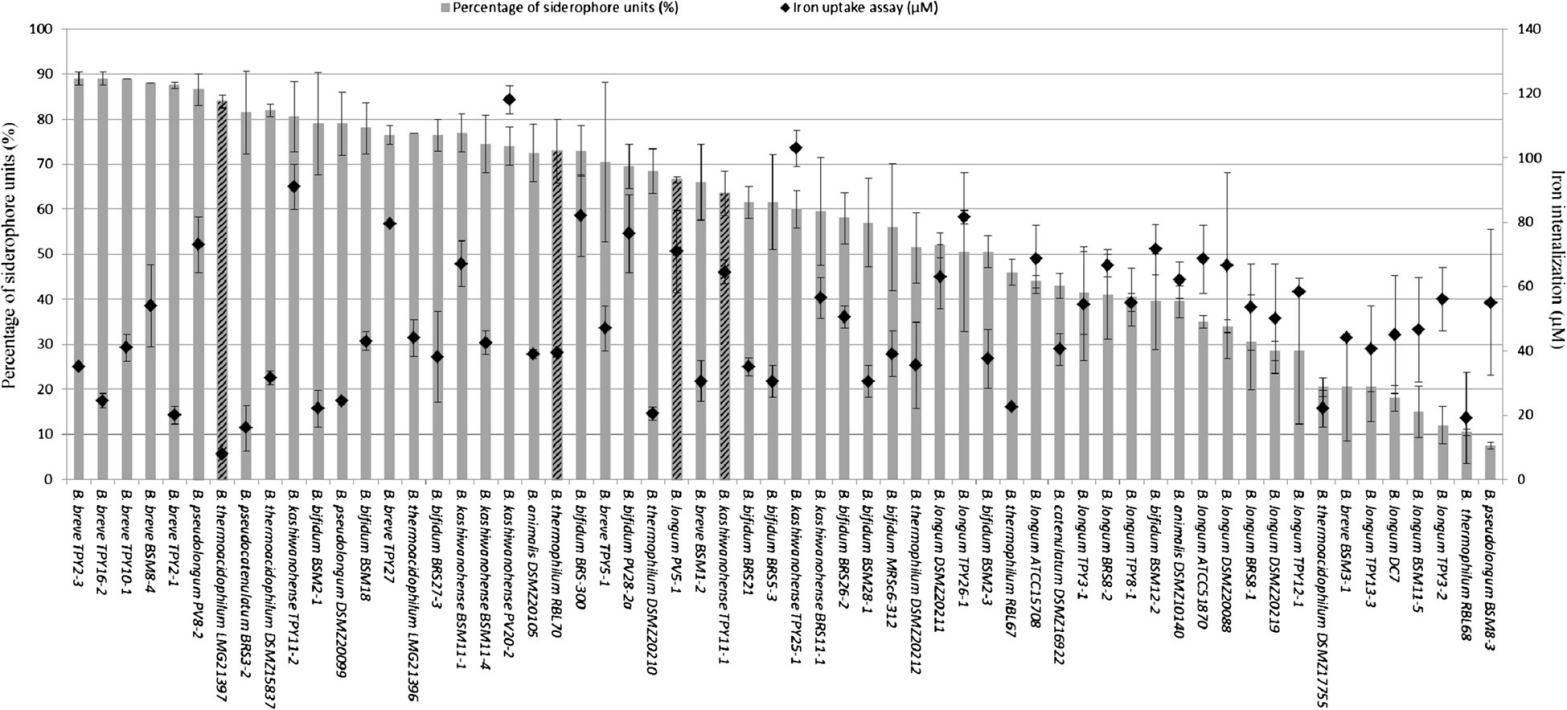
**Characterization of bifidobacterial strains for siderophore activity and iron uptake measured by the CAS assay and iron internalization test, respectively.** Columns show siderophore activity (%) (n = 2) and squares show the results of iron internalization (μM) (n = 2). Striped columns indicate selected strains after strain characterization. Error bars correspond to standard deviations calculated for two independent replicates for iron uptake and siderophore activity respectively.

Sixty bifidobacterial strains comprising 41 isolates and 19 culture collection strains were characterized by the iron uptake assay determined by using graphite furnace atomic absorption spectrophotometer. Iron internalization (II) was found to be strain dependent and *B. kashiwanohense* was the bifidobacterial species with the highest overall iron internalization of 78 μM +/− 27 (n = 7) (Figure 1b). Iron concentrations tested in bifidobacterial pellets ranged from 8 μM to 118 μM (Figure 2). Eight *B. longum* strains, six *B. kashiwanohense,* three *B. bifidum*, one *B. pseudolongum* and one *B. breve* showed significantly higher II values (p < 0.0001) than *B. thermoacidophilum* LMG21397 exhibiting the lowest II of 8 μM.

## Discussion

In the present study, 56 bifidobacterial strains with high iron sequestration properties were isolated from fecal samples of iron deficient Kenyan infants, most of them anemic, using an iron depleted culture dependent approach. We assumed for our research that in breast-fed infants little iron enters the colon and thus low iron availability is expected in the gut. Indeed, iron content in breast milk was reported to be in the range from 0.4 mg/L to 0.5 mg/L [25-27]. Thus, a high competition of strains for iron would be present in the gut of breast fed infants, creating selective pressure for strains with high iron sequestration properties. After the genotypic identification the iron sequestration properties of the isolates were measured and compared to those of culture collection strains.

Using qPCR targeting the *xfp* gene, high populations of bifidobacteria were found in fecal samples of Kenyan infants in agreement with previous studies where population was found to be in the range of 10^9^-10^10^ gene copy numbers per gram feces in breast fed infants [28,29]. To perform the enrichment of bifidobacterial diversity with high iron sequestration mechanisms a culture dependent approach was applied, combining the low iron environment characterized from Kenyan infants, with the use of 2,2-dipiridyl which is a strong chelator for iron. The most frequently isolated bifidobacterial species were *B. bifidum*, *B. longum*, *B. breve*, *B. pseudolongum* and *B. pseudocatenulatum* which are species frequently found in the infant gut [5,30]. The application of strong iron limitation during the isolation process may have limited the growth of some bifidobacterial species that could not grow under these stringent conditions. On the other hand the identification of strains from six phylogenetic taxa present in infant stool suggest that bifidobacteria is able to efficiently overcome iron limitation as one of the mechanisms active in the gut.

Using culture independent methods Turroni *et al.* [3] showed that a broad diversity of species were present in the gut of European infants. Major bifidobacterial groups identified were *B. breve*, *B. bifidum*, *B. longum*, *B. pseudocatenulatum* and *B. pseudolongum* in agreement with our study. As shown in Table 1, the most frequently isolated species in the present study were *B. breve*, *B. bifidum* and *B. longum* which are bifidobacterial species highly encountered in breast fed infants. In addition, using culture dependent studies, Turroni *et al.* [31] and Matsuki *et al.* [32] showed that the most frequently isolated species in fecal samples from European infants were *B. longum*, *B. adolescentis* and *B. breve*, *B. longum*, respectively. This is in contrast to our study where *B. animalis* or *B. catenulatum* were not detected while *B. kashiwanohense* was isolated in three fecal samples, possibly reflecting species distribution between African and European infants, a bias resulting from the stringent iron limitation, or a non-identified species in previous studies. *B. kashiwanohense* has been reported for the first time from feces of a healthy Japanese infant in 2011 and is known to be close to *B. pseudocatenulatum* and *B. catenulatum*, that are species commonly found in the infantile gut [33]. In addition, *B. kashiwanohense* was only found in three samples which can be associated with an interindividual variability in species distribution which was previously shown for premature and term infants [5,34,35].

Iron is an essential micronutrient for most microorganisms and directly impacts the metabolism of microorganisms. Functions regulated by iron in bacteria are those involved in iron utilization or acquisition and two transport systems have been described in iron uptake pathways [11]. Bacteria secrete iron scavenging compounds, siderophores, to fulfill their iron requirement for growth. A high fraction (41%) of bifidobacterial strains were in the high SA group, with *B. breve* exhibiting the highest siderophore activity. In addition, the major fraction of the strains exhibiting high SA belonged to the isolates from infants stool samples, which highlights the importance of testing new isolates for this property. Previous studies demonstrated that bifidobacterial strains can inhibit competing organisms in an iron concentration dependant manner [9]. However, it was also recently reported that the growth of some bifidobacterial strains was not affected by the presence of iron chelators such as 2,2-dipiridyl and 8-hydroxyquinoline [36,37]. Although different attempts have been done to identify iron sequestration mechanisms no study demonstrated the production of iron scavenging compounds in culture supernatant. Production of iron scavenging compounds is a common strategy to compete in environments where iron is limited and activation of this mechanism could represent an advantage for bifidobacteria in the presence of competing bacteria of the gut microbiota.

Bifidobacterial strains were characterized towards iron internalization which is a potential mechanism to scavenge iron and to provide competition. Interestingly, *B. kashiwanohense* showed the highest II activity suggesting that *B. kashiwanohense* has efficient sequestration systems that may play a role in competing for iron in the intestine [16]. Thus, the efficient iron uptake systems of bifidobacteria, such as production of iron scavenging compounds and iron internalization, may be important from the point of view of nutritional immunity [36,37].

Despite high SA in *B. breve* species and II in *B. kashiwanohense* species were identified, standard deviation within each species group was very high, reflecting a remarkably strain specificity towards SA and II. Different SA and II properties between species and strains enable the selection of strains with the highest iron sequestration activities with possible competitive advantage when competing in the iron limited intestine. No correlation was found between siderophore activity and iron internalization (Spearman’s correlation: −0.22) implicating that these two properties are independently controlled, in agreement with previously reported studies where the two properties were not related [12,38]. In our study sixteen out of twenty strains that showed high SA and II are strains isolated from iron deficient infant stools. Four strains exhibited both high SA and II characteristics: *B. pseudolongum* PV8-2, *B. kashiwanohense* PV20-2, *B. bifidum* PV28-2a, *B. longum* PV5-1 (Figure 2). The mechanisms involved in iron acquisition have been shown to play an important role during enteric infection [39-41]. It has been recognized that most of the dominant colonizers of an environment have efficient iron scavenging systems and can inhibit the growth of other competing organisms by depriving them from iron [12]. Herein, selected strains could be further tested for the utilization of iron sequestration mechanisms for host protection in complex gut ecosystems where mechanisms involved in iron competition have been addressed to play an important role [39].

## Conclusions

A wide diversity of bifidobacterial strains with high iron sequestration properties were obtained from fecal samples of iron deficient Kenyan infants in a culture dependent approach targeting low iron environments. Bifidobacterial species from public culture collections and newly isolated strains were characterized for siderophore production in culture supernatant and iron internalization which enabled the selection of four strains isolated from infant stools with high iron sequestration mechanisms. The implication of an efficient utilization of iron sequestration mechanisms by selected bifidobacteria strains to inhibit enteric pathogens could be further tested in complex gut ecosystems and may uncover one of the mechanisms of bifidobacteria for host protection against infection.

## Methods

### Culture collection strains

Twenty-one *Bifidobacterium* strains belonging to different culture collections (DSMZ, ATCC, LMG) and nine belonging to our own culture collection were used for strain characterization and are listed in Table 3. Additionally, 56 bifidobacterial strains typed to species level were isolated from 28 stool samples from Kenyan infants (Table 2).

**Table 3:** **Strains from culture collections used for characterization of siderophore activity and iron internalization**

**Strain**	**Culture collection**	**Number**
*Bifidobacterium adolescentis*	DSMZ	20083
*Bifidobacterium animalis*	DSMZ	10140
*Bifidobacterium animalis*	DSMZ	20105
*Bifidobacterium bifidum*	DSMZ	20456
*Bifidobacterium boum*	DSMZ	20432
*Bifidobacterium breve*	DSMZ	20213
*Bifidobacterium catenulatum*	DSMZ	16992
*Bifidobacterium lactis*	LFB culture collection	DC1
*Bifidobacterium lactis*	LFB culture collection	DC5
*Bifidobacterium longum*	ATCC	15708
*Bifidobacterium longum*	ATCC	51870
*Bifidobacterium longum*	LFB culture collection	DC7
*Bifidobacterium longum*	LFB culture collection	NCC2705
*Bifidobacterium longum*	DSMZ	20088
*Bifidobacterium longum*	DSMZ	20211
*Bifidobacterium longum*	DSMZ	20219
*Bifidobacterium pseudocatenulatum*	DSMZ	20438
*Bifidobacterium pseudolongum*	DSMZ	20099
*Bifidobacterium thermoacidophilum*	DSMZ	15837
*Bifidobacterium thermoacidophilum*	DSMZ	17755
*Bifidobacterium thermoacidophilum*	LMG	21395
*Bifidobacterium thermoacidophilum*	LMG	21396
*Bifidobacterium thermoacidophilum*	LMG	21397
*Bifidobacterium thermophilum*	LFB culture collection	RBL67
*Bifidobacterium thermophilum*	LFB culture collection	RBL68
*Bifidobacterium thermophilum*	LFB culture collection	RBL70
*Bifidobacterium thermophilum*	DSMZ	20210
*Bifidobacterium thermophilum*	DSMZ	20212
*Bifidobacterium thermophilum*	LFB culture collection	F9K9
*Bifidobacterium spp.*	LFB culture collection	BB46

DSMZ: German Collection of Microorganisms, ATCC: American Type Culture Collection, LMG: Belgian co-ordinated Collections of Micro-organisms, LFB: Laboratory of Food Biotechnology ETH Zurich culture collection.

### Cultivation conditions

Working cultures were obtained by inoculating strain stocks (1% inoculation rate) maintained at −80°C in 10 mL de Man, Rogosa and Sharpe broth (Biolife, Italy) (MRS), pH 6.0, supplemented with 0.5 g/L L-cysteine (cys) (Sigma-Aldrich, Switzerland). Incubation was carried out in an anaerobic chamber (Coy Laboratory Products Inc., Inc.) with an atmosphere of 85% N_2_, 10% CO_2_ and 5% H_2_ (PanGas AG, Switzerland) at 37°C for 48 h. Peptone water (Oxoid, Switzerland) supplemented with 0.05% cys, pH 6.5 (peptone-cys) was used as a re-suspension solution. In order to achieve iron limited conditions a chemically semi-defined low iron medium (CSDLIM, tested with 1.5 μM iron) was used, containing: 5.64 g/L 5X M9 minimal salts, 5.0 g/L proteose-peptone (Becton Dickinson, Switzerland), 0.2 g/L cys, 20 mL/L of 20% glucose, 2 mL/L of 1 M MgSO_4_ and 0.2 mL/L of 1 M CaCl_2_ 2H_2_O (Sigma-Aldrich, Switzerland). The M9 minimal salts, proteose-peptone and cys were dissolved in 980 mL of double distilled water and autoclaved. Glucose, magnesium and calcium were solubilized in double distilled water to the desired concentration (see above), filter-sterilized (0.22 μm pore size, Millipore, Switzerland), and added to the autoclaved portion. For measuring the ability to internalize iron an iron uptake solution (IUS), with 0.05 μM Fe, pH 6.5 was used, containing: 0.4 g/L KCl; 8 g/L NaCl; 0.14 g/L CaCl_2_ 2H_2_O; 2 g/L glucose; 8.2 g/L sodium acetate (Sigma-Aldrich, Switzerland); 0.2 g/L cys was used.

### Fecal samples for *Bifidobacterium* strains isolation

Twenty-eight baseline stool samples from Kenyan infants (fifteen male, thirteen female) were used for *Bifidobacterium* strain isolation [42]. Stools were obtained from eighteen anemic and six iron deficient infants (hemoglobin level <11 g/dL) and from four infants for whom no hemoglobin data was available. All infants were 6 +/−0.25 months old, vaginally delivered, breast fed, unrelated, and they did not receive any antibiotic/probiotic treatment in the previous three months. Stool samples of approx. 1 g of fresh fecal material were taken up with a sterile spatula and collected in cryo-vials containing 20% glycerol (Sigma-Aldrich, Switzerland) and 0.05% (final volume) cys (Sigma-Aldrich, Switzerland). The samples were frozen at −20°C until processing for isolation. This study was approved by the Ethics and Research Committees of the Kenyatta National Hospital/ University of Nairobi (KNH-ERC/A/337), the University of KwaZulu-Natal (BF121/08) and the Swiss Federal Institute of Technology Zurich (EK 2009-N-53). Caregivers signed an informed consent. This study is registered at clinicaltrials.gov as NCT01111864.

### Characterization of fecal samples

#### DNA extraction and quantitative PCR (qPCR)

DNA was extracted from 250 +/− 50 mg fecal sample by a FastDNA SPIN kit for soil (MP Biomedicals, France) and quantification was carried out with a Nanodrop Spectrophotometer (ND-1000, Witec AG, Switzerland). Total bacteria and bifidobacteria populations were detected by qPCR with KAPA™ SYBR® FAST qPCR kit (Biolabo Scientifics Instruments SA, Switzerland) and a 7500 Fast Real-Time PCR System (Applied Biosystems, Switzerland). Total bacteria were quantified using plasmid pLME21 containing the 16S rRNA gene from *Escherichia coli* JM109 which was amplified with the following primers, Eub338 F (5′-ACTCCTACGGGAGGCAGCAG-3′) and Eub518R (5′-ATTACCGCGGCTGCTGG-3′) [22]*. Bifidobacterium* spp. was quantified by targeting the xylulose-5-phosphate/fructose-6-phosphatephosphoketolase gene (*xfp*) with the following primers, xfp-fw (5′-ATC TTCGGACCBGAYGAGAC-3′) and xfp-rv (5′-CGATVACGTGVACGAAGGAC-3′) [43,44]. Samples were analyzed twice in a total volume of 25 μL using KAPA™SYBR®FAST qPCR Master Mix (24 μL) containing 1 μL template DNA diluted 1:100 and 1:10 for total bacteria and bifidobacteria, respectively. Sample mean gene copy numbers (CN) per gram of feces (n = 2) were obtained from standard curves generated for each qPCR run using serial dilutions of control standard amplicons. PCR cycles consisted of initial activation at 95°C for 10 min, 40 cycles of denaturation at 95°C for 15 s, annealing at 60°C for 30 s, and elongation at 60°C for 30 s.

### Strain isolation

Stool samples were streaked on Beerens agar (BRS) [45]; de Man, Rogosa and Sharpe agar (Biolife, Italy) (MRS), supplemented with 0.5 g/L L-cysteine HCl (cys) (Sigma-Aldrich, Switzerland), pH 6.0; Trypticase-phytone-yeast (TPY) [46] agar and Bifidus selective agar (BSM) (Fluka, Switzerland) [47].To enhance the isolation of bifidobacteria with high iron sequestration properties a strong iron chelator, 2,2-dipyridyl (Sigma-Aldrich, Switzerland) at 50 μM [48] was supplemented to the enrichment agar. Incubation was carried out in the anaerobic chamber at 37°C for 48 h. Based on different morphologies three colonies were picked per enrichment agar, streaked for purity and cultured in MRS-cys broth. Cultures were grown to an OD_600nm_ (Biowave CO8000, Biochrom, England) of 1 +/− 0.5. Purity was verified microscopically and cultures were stored at −80°C in a final concentration of 20% (vol/vol) glycerol and 0.05% cys. Pellets obtained from liquid cultures were stored at −20°C for DNA extraction.

### Genotypic characterization

DNA was extracted from cell pellets using a Wizard Genomic DNA purification kit (Promega AG, Switzerland) according to the manufacturer’s instructions and strains were typed to species level using 16S rRNA sanger sequencing as described by Jost *et al.*, 2013 [49]. Briefly, PCR amplification of 16S rRNA genes was performed using a 4:1 mixture of forward primers 8f (5′-AGAGTTTGATCMTGGCTC AG-3′, universal) and 8f-bif (5′-AGGGTTCGATTCTGGCTCAG-3′, *Bifidobacterium*-specific) and a universal bacterial reverse primer 1391R (5′-GACGGGCGGTGTGTRCA-3′) (Microsynth AG, Switzerland). PCR reaction mixture of 50 μL contained 25 μL of 2X MasterMix (Fermentas GmbH, Switzerland), 0.2 μM of each primer (−mixture) and 1 μl of template DNA diluted to 1 ng/μL. Thermocycling (BiometraTProfessional Thermocycler; Biolabo Scientific Instruments SA, Switzerland) was performed with an initial denaturation step at 94°C for 300 s, a second denaturation step was carried out by thirty cycles at 94°C for 30 s, annealing at 57°C for 60 s and extension at 72°C for 30 s with a final extension at 72°C for 420 s. Specificity and amplicon size were verified by electrophoresis in 1.5% (w/v) agarose gels, and reactions were purified using an illustra GFX PCR DNA and Gel Band Purification Kit (GE Healthcare Europe GmbH, Switzerland) according to the manufacturer’s instructions. Cycle sequencing PCR was carried out in 20 μL reaction volumes with 5% (v/v) BigDye v3.1 (Applied Biosystems Europe BV), 4 μL of 5X sequencing buffer (Applied Biosystems, Switzerland), 1 μM of reverse primer 1391R and 1 μL of purified PCR template. Thermocycling (labcycler; SensoQuest GmbH, Switzerland) was performed with an initial denaturation step at 96°C for 300 s, followed by thirty-five cycles of denaturation at 96°C for 10 s, annealing at 55°C for 20 s and extension at 60°C for 240 s. Reactions were purified by dextran gel bead filtration (Sephadex; GE Healthcare, Switzerland) before loading 10 μL for capillary electrophoresis (ABI 3130xl DNA Analyzer; Applied Biosystems, Switzerland). Sequencing trace chromatograms were quality-trimmed and checked for miscalled bases using CLC Genomics Workbench 64 v6.0.5 (CLC bio, Denmark). DNA extraction and Sanger sequencing were carried out twice from two independent cultures. 16S rRNA partial sequences were aligned with the Basic Local Alignment Search Tool algorithm (BLAST) to the GenBank database and genotypic assignments were based on the nearest neighbor (<97% sequence similarity).

### Siderophore production

Eighty-six bifidobacteria strains from isolation and culture collections and were tested for siderophore production in culture supernatant by the Chroma Azurol S (CAS) assay [15]. Cells were grown twice in 10 mL MRS-cys broth under anaerobic conditions, centrifuged 10,000 *g* for 10 min at 24°C, supernatant discarded and re-suspended in peptone-cys water. CSDLIM medium (15 mL) was inoculated with 2% v/v of bacterial re-suspension. Strains were cultured anaerobically to an OD_600nm_ of 1.5 +/− 0.3 corresponding to log 6 +/− 0.3 CFU/mL. Siderophore activity was measured in culture supernatant and calculated as percentage of siderophore units according to Payne *et al.* [48]. Briefly, 2 mL of culture were centrifuged at 10,000 *g* for 10 min at 4°C. 0.5 mL of cultured supernatant was mixed with 0.5 mL of CAS dye and 0.01 mL of shuttle solution and absorbance (As) was measured at 630 nm (OD_630nm_) after 6 hours. 0.5 mL of uncultured media plus 0.5 mL of CAS dye and 0.01 mL of shuttle solution were mixed to obtain the absorbance reference (Ar) and uncultured CSDLIM media served as blank for absorbance measurements. A formula was used to obtain percentage of siderophore units: % of siderophore units = [(Ar – As)/Ar] x 100. *L. casei* DSMZ 20011 was used as negative control since according to Pandey [10] siderophores are not synthesized by *Lactobacillus* spp.. *E. coli* K12 was used as positive control due to their ability to produce siderophores in low iron environments [50]. From the starting 86 bifidobacterial strains tested isolates showing similar (97-100%) genotypic identification in the 16 s rRNA, isolated from same stool sample and plate, and that also show similar SA value; as well as, strains showing low siderophore activity, were excluded for the iron uptake (II) assay.

### DNA finger printing

Phylogenetic diversity of sixty-two *Bifidobacterium* strains included in the II, was evaluated by DNA-finger printing using RAPD and REP-PCR. RAPD reactions were performed according to Jost *et al.* [51]. RAPD amplification reactions consisted of 25 μL that contained 12.5 μL of 2X Master Mix (Fermentas, Switzerland), 1 μM of primer (100 μM) (Mycrosynth, Switzerland) and 1 μL of DNA template diluted to approximately 50 ng/μL. Four separate RAPD reactions were performed for each species, using primers OPA-02 (5′-TGCCGAGCTG-3′), OPL-07 (5′-GGGAACGTGT-3′), OPL-16 (5′-GGGAACGTGT-3′) and OPA-13 (5′- CAGCACCCAC-3′). For reactions using primers OPA-02, OPL-07, OPL-13 and OPL-16, thermocycling was performed with an initial denaturation step at 95°C for 3 min, followed by 45 cycles of denaturation at 94°C for 1 min, annealing at 30°C for 1 min and extension at 72°C for 2 min. Due to the limited number of bands identified in *B. kashiwanohense* and *B. pseudolongum* species, REP-PCR was performed as described previously [52]. REP-PCR was performed using the primer REP1R-I (5′-NNNNCGNCGNCATCNGGC-3′) and REP2-I (5′-NCGNCTTATCNGGCCTAC-3′) (Mycrosynth, Switzerland). REP-PCR reactions were carried out with an initial denaturation step at 95°C for 3 min, followed by 30 cycles at 90°C for 30 s, 40°C for 1 min and 72°C for 1.5 min, and final extension at 72°C for 8 min. RAPD and REP-PCR products were visualized by UV light on ethidium bromide stained agarose (1.5%) after gel electrophoresis in 1 × TAE-buffer (pH 8.0), using 1-kb ladder (Fermentas GmbH, Switzerland) as reference.

### Characterization based on iron internalization

Sixty strains, 41 isolates and 19 strains belonging to culture collections, were characterized for iron uptake characterization. For iron internalization the method described by Kot *et al.* [53] was used with some modifications. Briefly, *Bifidobacterium* strains were grown twice under anaerobic conditions in MRS-cys broth for 24 h at 37°C. Cultures were centrifuged 10,000 *g* (Biofuge primo, Heraeus, Switzerland) at 24°C, 10 min and supernatant discarded. A cell suspension was prepared with OD_600nm_ adjusted to 1 with peptone-cys water. This suspension was used to inoculate 25 mL of CSDLIM medium at 2% v/v and cultured to an OD_600nm_ of 1 +/− 0.25. Culture was centrifuged and supernatant discarded, and cells were resuspended in 10 mL volume of the iron uptake solution (IUS) to yield a concentration of log 8+/− 0.3 CFU/mL. Two tubes of same volume containing the standardized culture were prepared to determine the amount of iron internalization. The first tube without added iron served as the reference for the original amount of iron present in the pellet. The second tube was supplemented with 35 μM FeSO_4_ freshly prepared and was used to measure the amount of internalized iron. Both tubes were incubated for 60 min at 37°C under anaerobic conditions, then placed immediately on ice, centrifuged 15 min at 10,000 *g* (Biofuge primo, Heraeus), and washed twice with 2 mL sterile double distilled water. All samples were processed in duplicate. Iron concentration in the pellet (μM) was measured by graphite furnace atomic absorption spectrophotometer (AA-240Z, Varian Inc., Australia) according to manufacturer’s instructions. Briefly, pellet was mixed with, 0.5 mL of nitric acid 65% added with 0.5 mL of ultrapure water (Super-Q® plus, Millipore, Switzerland), sonicated for 30 min at room temperature, and centrifuged 5 min, 10,000 *g*. Supernatants were diluted with ultrapure water to adequate iron concentration. Standard curves were generated by serial dilution of a commercial iron standard solution (Titrisol, Merck Chemicals, Germany).

### Statistical analysis

Data from two independent trials were analyzed by comparing the means with the nonparametric, multiple comparisons with control, Dunnett’s method. For statistical analysis of siderophore activity, *B. bifidum* MRSc6-292 served as control strain with the lowest siderophore activity and was compared to the rest of the strains. Control sample for statistical analysis of iron internalization was *B. thermoacidophilum* LMG21397 with the lowest iron internalization value. Correlation between the two set of data was assessed by Spearman’s correlation.

### Nucleotide accession numbers

The main nucleotide sequences determined in this study have been assigned GenBank Accession numbers: [GenBank: KJ412975-KJ412985].

## Additional files

Additional file 1: Figue S1 Characterization of eighty-six bifidobacterial strains towards siderophore activity measured by the CAS assay and columns show siderophore activity (%) (n = 2). Characterization of eighty-six bifidobacterial strains towards siderophore activity measured by the CAS assay and columns show siderophore activity (%) (n = 2). Error bars correspond to standard deviations calculated for two independent replicates for siderophore activity. Light gray columns correspond to strains belonging to public culture collections (DSMZ, ATCC, LMG). Dark gray columns correspond to isolates from stool samples of Kenyan infants. Additional file 2: Table S1 Identity and siderophore activity of bifidobacterial strains isolated from stool of Kenyan infant of different iron status. Additional file 3: Figue S2 RAPD and REP fingerprints of *Bifidobacterium* spp. isolated from stool of breast fed Kenyan infants included in the characterization iron internalization using a) OPA-02 primer, b) OPL-07 primer, c) OPL-13 primer, d) OPL-16 primer for RAPD-PCR and REP1R-I primer, REP2-I primer for REP-PCR. RAPD and REP fingerprints of *Bifidobacterium* spp. isolated from stool of breast fed Kenyan infants included in the characterization of SA and II using a) OPA-02 primer, b) OPL-07 primer, c) OPL-13 primer, d) OPL-16 primer for RAPD-PCR and REP1R-I primer, REP2-I primer for REP-PCR.
