# Supplementary figures and images for: Bifidobacteria strains isolated from stools of iron deficient infants can efficiently sequester iron
Source: BMC Microbiol. 2015 Jan 16;15(1):3. doi: 10.1186/s12866-014-0334-z (PMC4320568; doi:10.1186/s12866-014-0334-z)

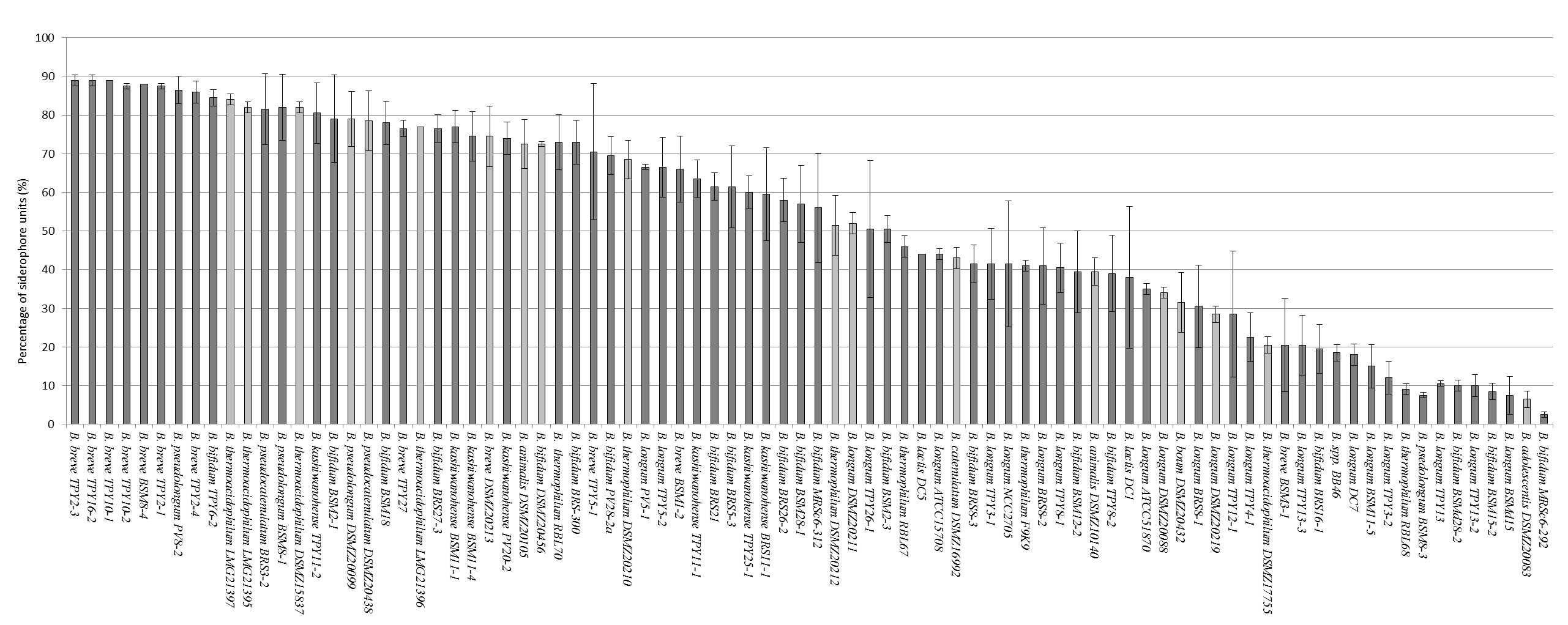


Figue S1

Supplement: Additional file 1: Figue S1 — Characterization of eighty-six bifidobacterial strains towards siderophore activity measured by the CAS assay and columns show siderophore activity (%) (n = 2). Characterization of eighty-six bifidobacterial strains towards siderophore activity measured by the CAS assay and columns show siderophore activity (%) (n = 2). Error bars correspond to standard deviations calculated for two independent replicates for siderophore activity. Light gray columns correspond to strains belonging to public culture collections (DSMZ, ATCC, LMG). Dark gray columns correspond to isolates from stool samples of Kenyan infants. [file 12866_2014_334_MOESM1_ESM.doc]

## Slide 1
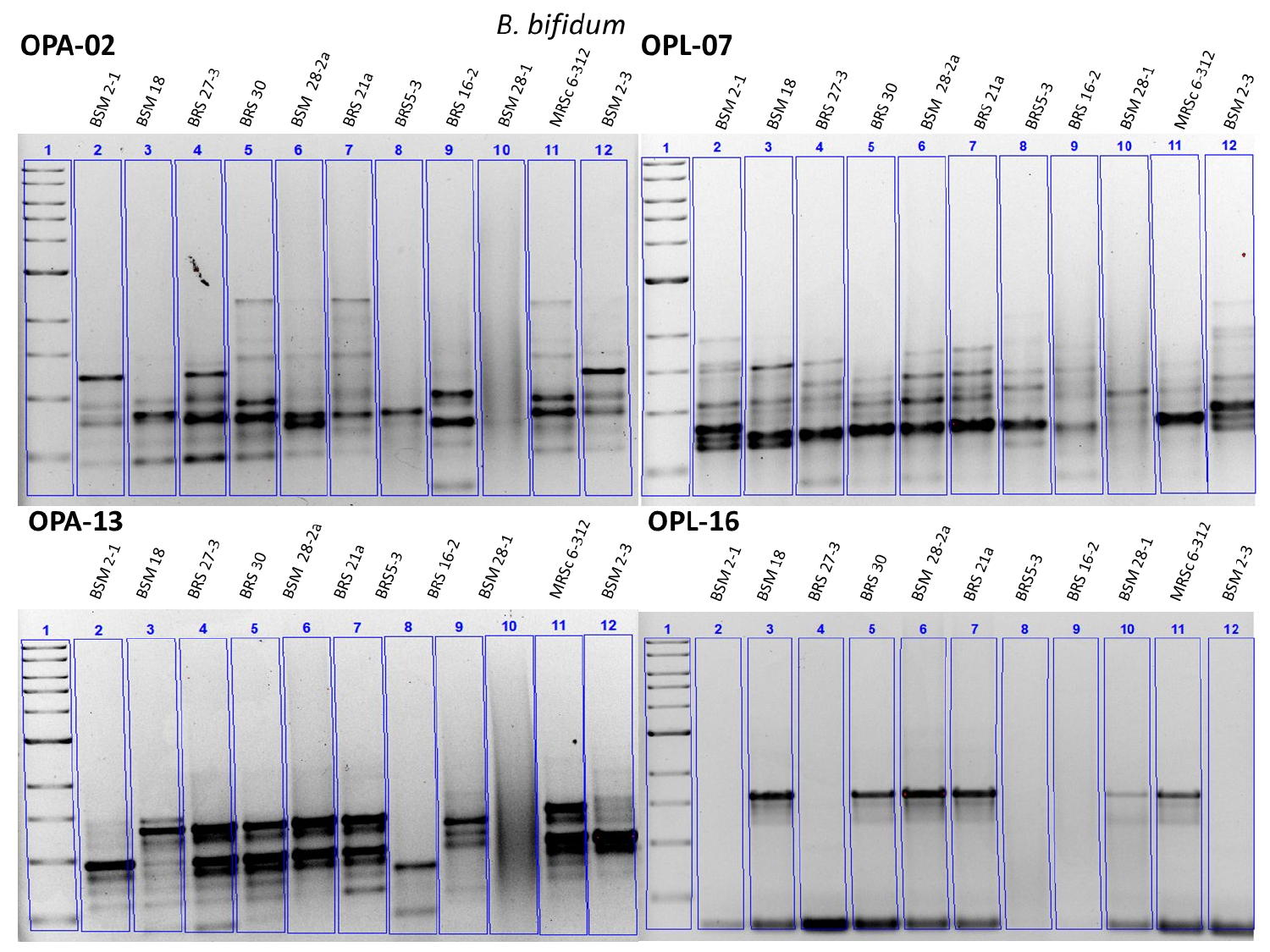

## Slide 2
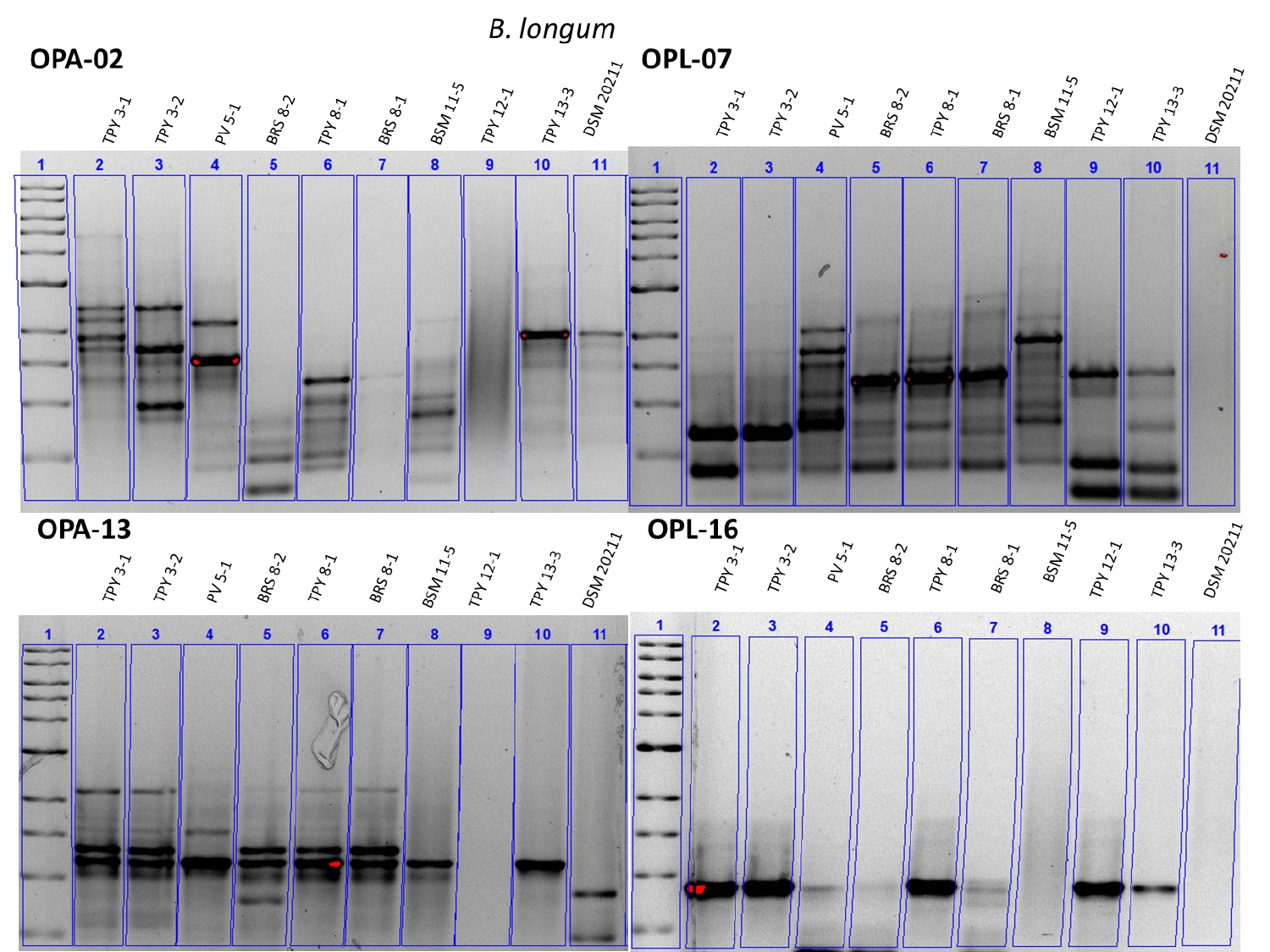

## Slide 3
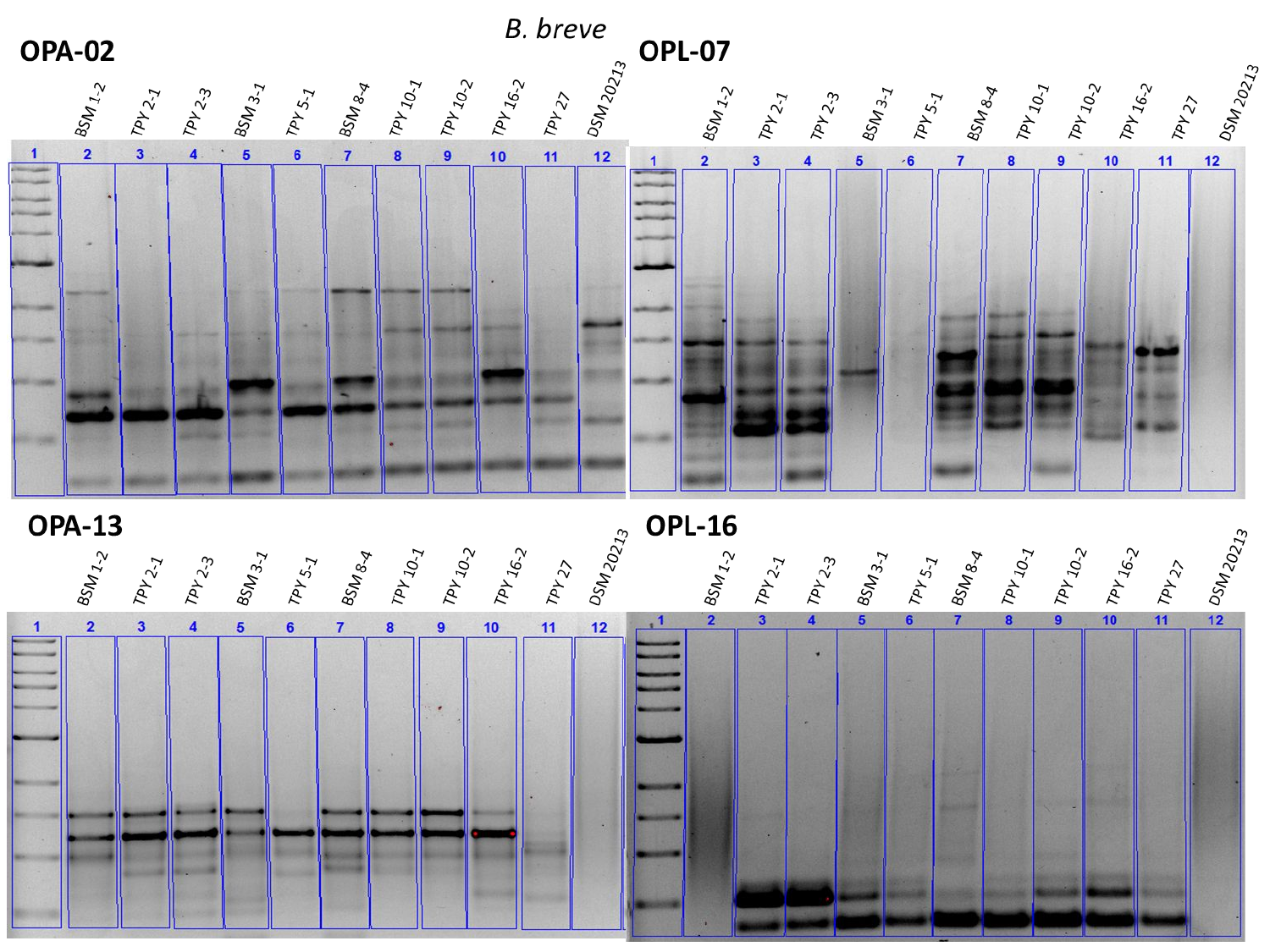

## Slide 4
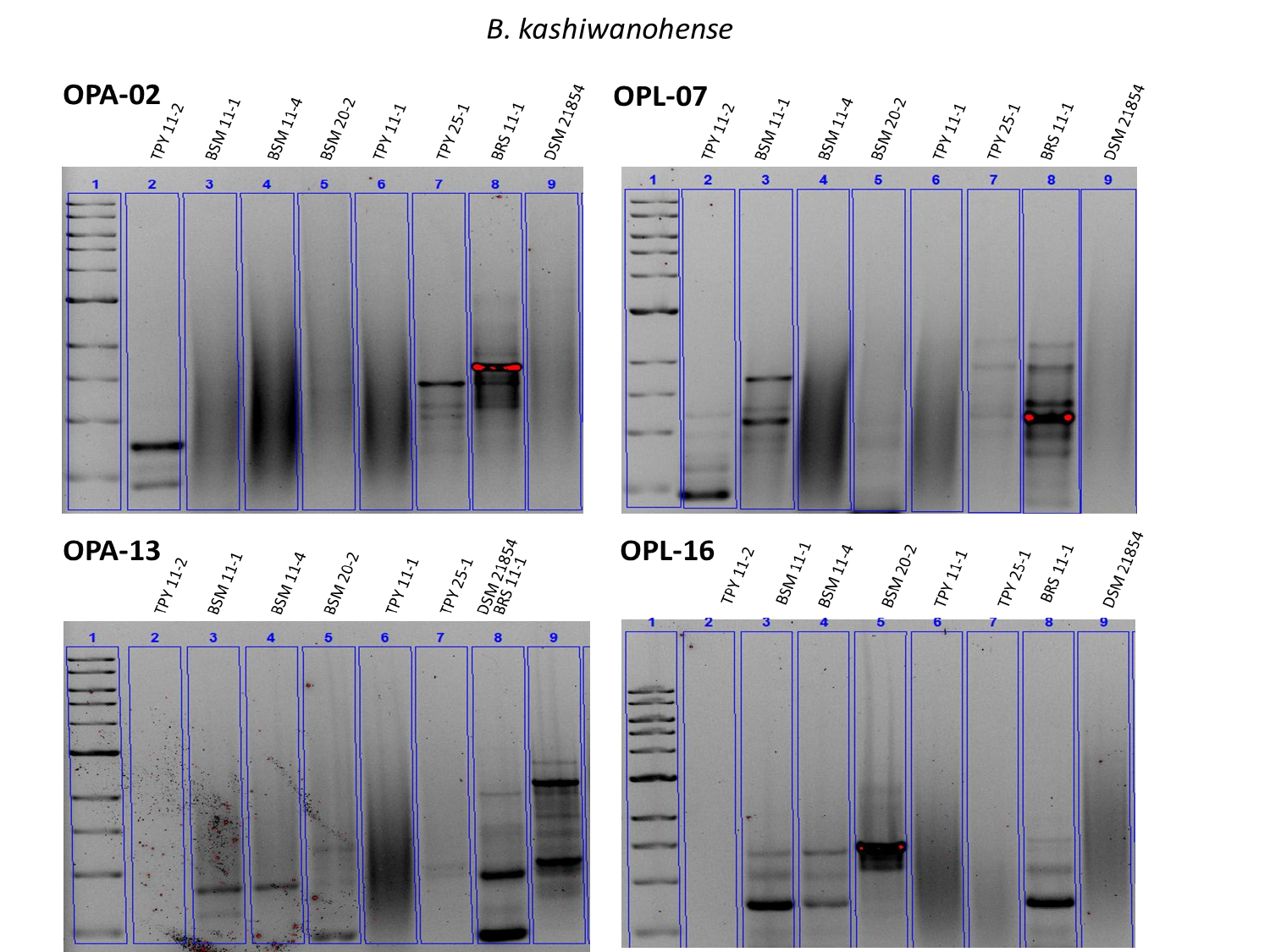

## Slide 5
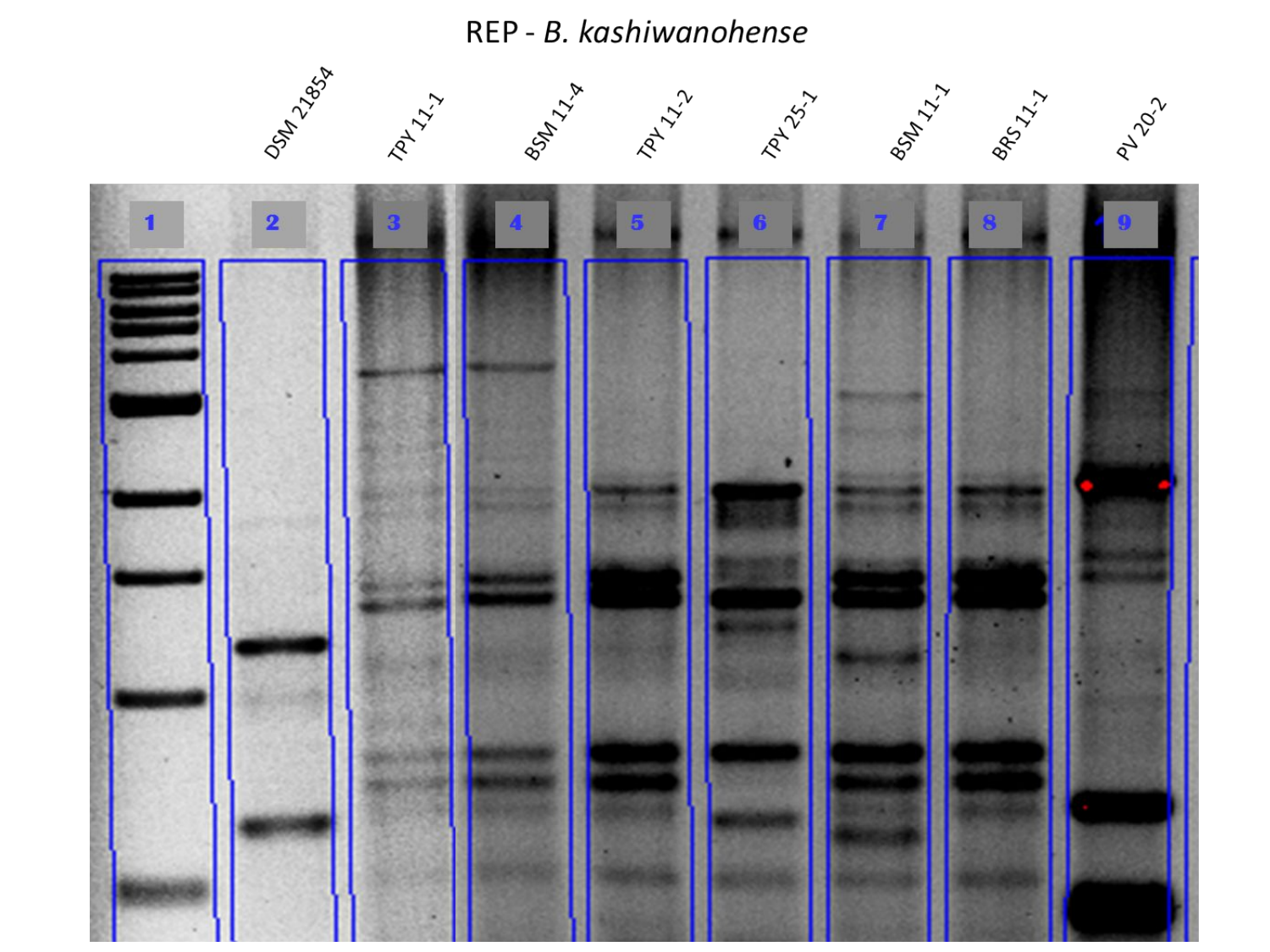

## Slide 6
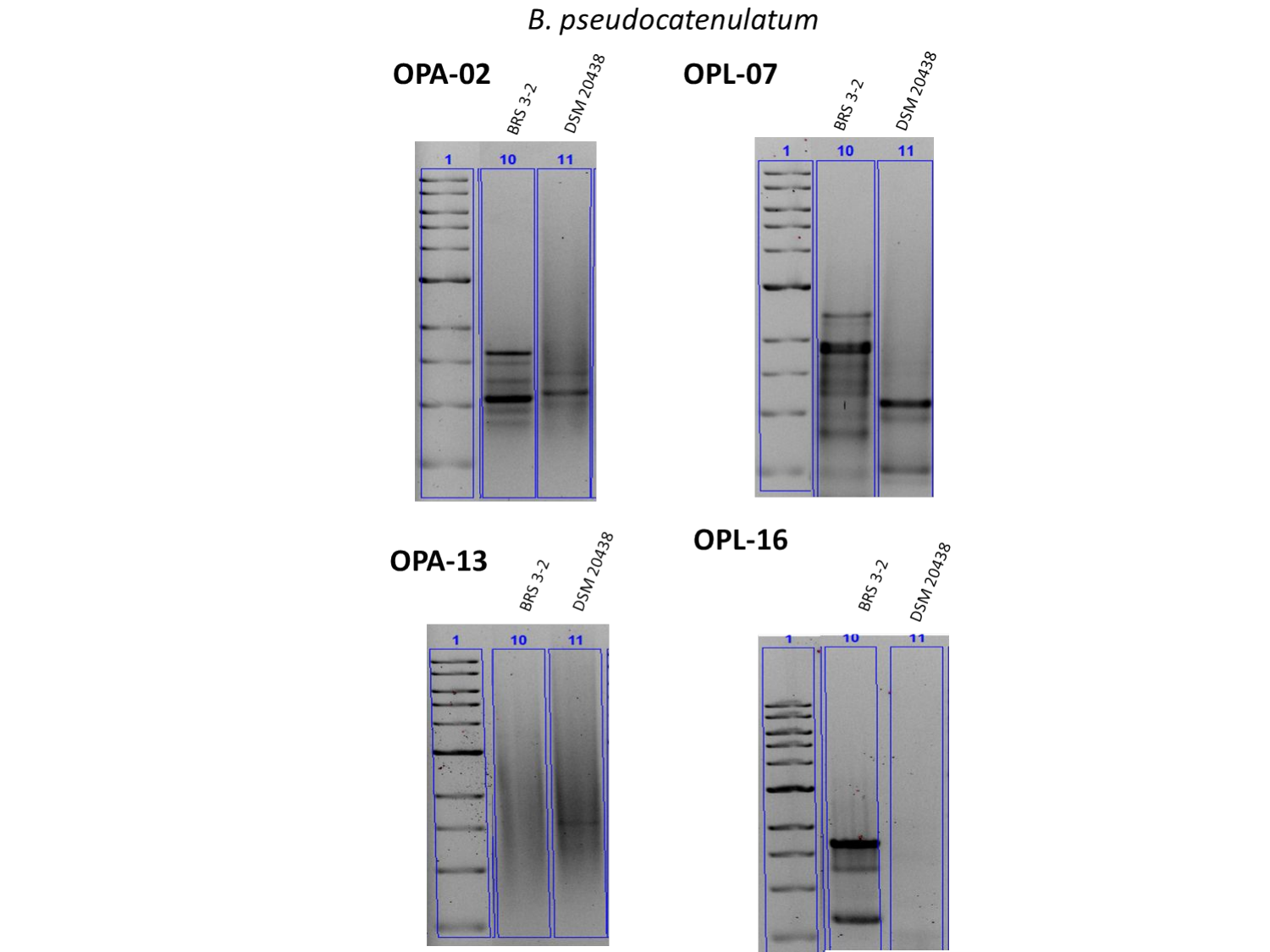

## Slide 7
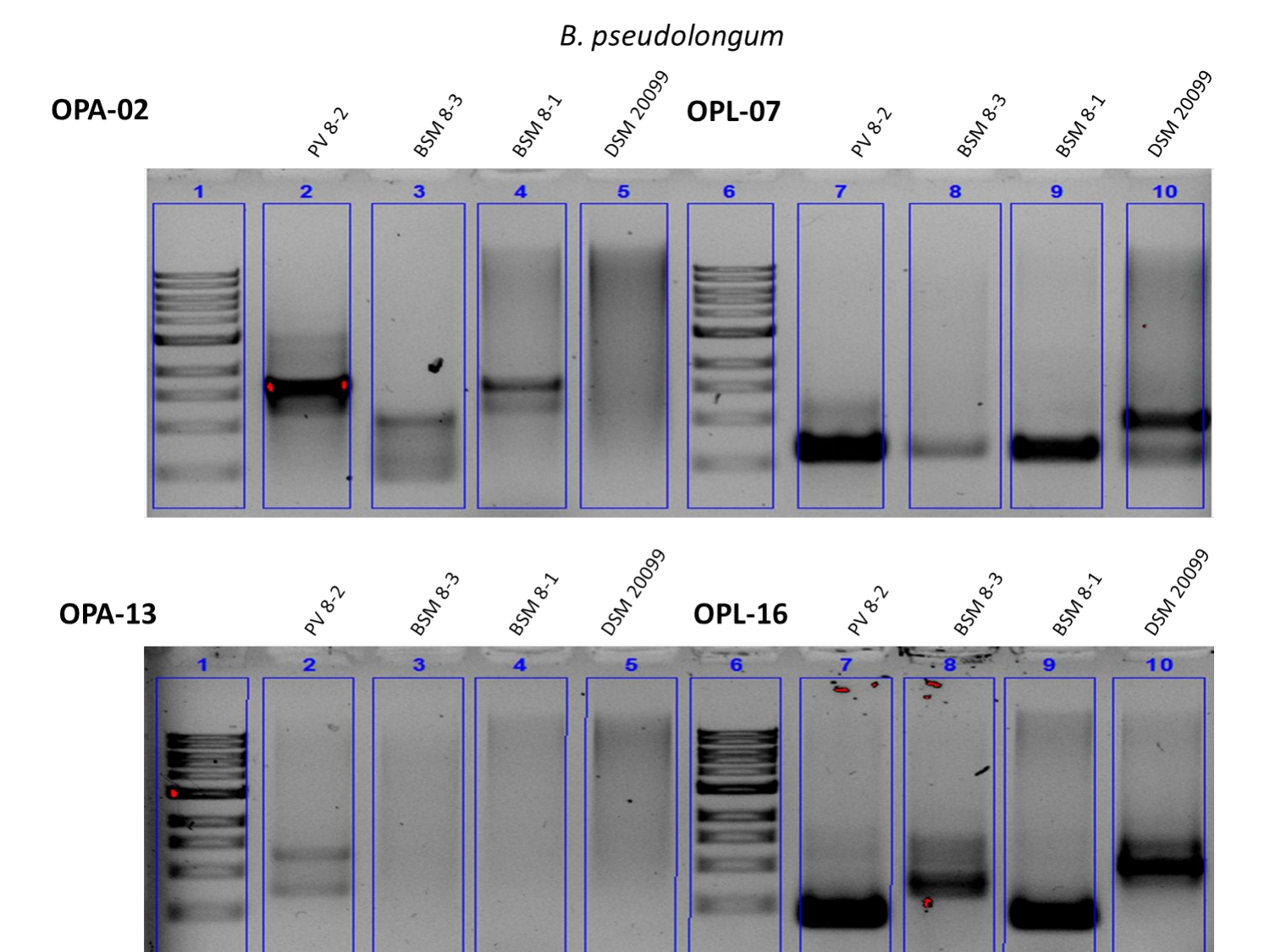

## Slide 8
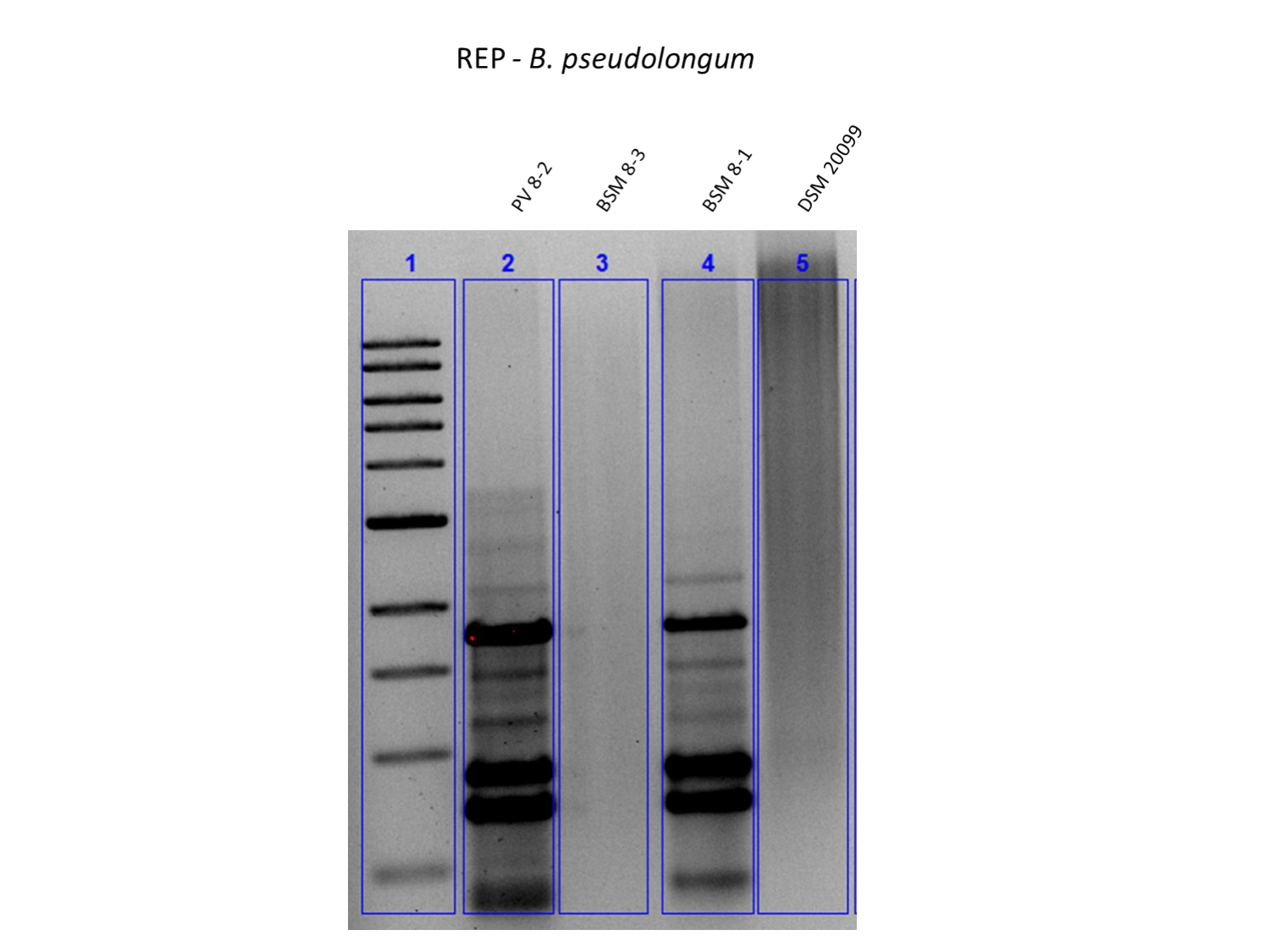

Supplement: Additional file 3: Figue S2 — RAPD and REP fingerprints of Bifidobacterium spp. isolated from stool of breast fed Kenyan infants included in the characterization iron internalization using a) OPA-02 primer, b) OPL-07 primer, c) OPL-13 primer, d) OPL-16 primer for RAPD-PCR and REP1R-I primer, REP2-I primer for REP-PCR. RAPD and REP fingerprints of Bifidobacterium spp. isolated from stool of breast fed Kenyan infants included in the characterization of SA and II using a) OPA-02 primer, b) OPL-07 primer, c) OPL-13 primer, d) OPL-16 primer for RAPD-PCR and REP1R-I primer, REP2-I primer for REP-PCR. [file 12866_2014_334_MOESM3_ESM.ppt]
