# Supplementary material for: Bifidobacteria strains isolated from stools of iron deficient infants can efficiently sequester iron
Source: BMC Microbiol. 2015 Jan 16;15(1):3. doi: 10.1186/s12866-014-0334-z (PMC4320568; doi:10.1186/s12866-014-0334-z)
Supplement: Additional file 2: Table S1 — Identity and siderophore activity of bifidobacterial strains isolated from stool of Kenyan infant of different iron status. [file 12866_2014_334_MOESM2_ESM.docx]

**Table S1. Identity and siderophore activity of bifidobacterial strains isolated from stool of Kenyan infant of different iron status.**

| **Fecal sample** | **Strain ID** | **Iron status** | **Siderophore value (Average n=2)** | **SDV** |
| --- | --- | --- | --- | --- |
| 1 | *B. breve* BSM1-2 | anemic | 66 | 8 |
| 2 | *B. breve* TPY2-1 | anemic | 88 | 1 |
| 2 | *B. breve* TPY2-3 | anemic | 89 | 1 |
| 2 | *B. breve* TPY2-4 | anemic | 86 | 3 |
| 2 | *B. bifidum* BSM2-1 | anemic | 79 | 11 |
| 2 | *B. bifidum* BSM2-3 | anemic | 51 | 4 |
| 3 | *B. pseudocatenulatum* BRS3-2 | anemic | 82 | 9 |
| 3 | *B. bifidum* BRS-300 | anemic | 73 | 6 |
| 3 | *B. longum* TPY3-1 | anemic | 42 | 9 |
| 3 | *B. longum* TPY3-2 | anemic | 12 | 4 |
| 3 | *B. breve* BSM3-1 | anemic | 21 | 12 |
| 4 | *B. longum* TPY4-1 | anemic | 23 | 6 |
| 5 | *B. longum* PV5-1 | anemic | 67 | 1 |
| 5 | *B. breve* TPY5-1 | anemic | 71 | 18 |
| 5 | *B. longum* TPY5-2 | anemic | 67 | 8 |
| 5 | *B. bifidum* BRS5-3 | anemic | 62 | 11 |
| 6 | *B. bifidum* TPY6-2 | anemic | 85 | 2 |
| 6 | *B. bifidum* MRSc6-292 | anemic | 3 | 1 |
| 6 | *B. bifidum* MRSc6-312 | anemic | 56 | 14 |
| 8 | *B. pseudolongum* PV8-2 | anemic | 87 | 4 |
| 8 | *B. pseudolongum* BSM8-1 | anemic | 82 | 8 |
| 8 | *B. psedolongum* BSM8-3 | anemic | 8 | 1 |
| 8 | *B. breve* BSM8-4 | anemic | 88 | 0 |
| 8 | *B. longum* BRS8-2 | anemic | 41 | 10 |
| 8 | *B. bifidum* BRS8-3 | anemic | 42 | 5 |
| 8 | *B. longum* TPY8-1 | anemic | 41 | 6 |
| 8 | *B. bifidum* TPY8-2 | anemic | 39 | 10 |
| 8 | *B. longum* BRS8-1 | anemic | 31 | 11 |
| 10 | *B. breve* TPY10-1 | anemic | 89 | 0 |
| 10 | *B. breve* TPY10-2 | anemic | 88 | 1 |
| 11 | *B. kashiwanohense* TPY11-1 | anemic | 64 | 5 |
| 11 | *B. kashiwanohense* TPY11-2 | anemic | 81 | 8 |
| 11 | *B. kashiwanohense* BSM11-1 | anemic | 77 | 4 |
| 11 | *B. kashiwanohense* BSM11-4 | anemic | 75 | 6 |
| 11 | *B. longum* BSM11-5 | anemic | 15 | 6 |
| 11 | *B. kashiwanohense* BRS11-1 | anemic | 60 | 12 |
| 12 | *B. longum* TPY12-1 | iron deficient | 29 | 16 |
| 12 | *B. bifidum* BSM12-2 | iron deficient | 40 | 11 |
| 13 | *B. longum* TPY13 | anemic | 11 | 1 |
| 13 | *B. longum* TPY13-2 | anemic | 10 | 3 |
| 13 | *B. longum* TPY13-3 | anemic | 21 | 8 |
| 15 | *B. bifidum* BSM15-2 | anemic | 9 | 2 |
| 15 | *B. longum* BSMd15 | anemic | 8 | 5 |
| 16 | *B. breve* TPY16-2 | anemic | 89 | 1 |
| 16 | *B. bifidum* BRS16-1 | anemic | 20 | 6 |
| 18 | *B. bifidum* BSM18 | anemic | 78 | 6 |
| 20 | *B. kashiwanohense* PV20-2 | iron deficient | 74 | 4 |
| 21 | *B. bifidum* BRS21 | iron deficient | 62 | 4 |
| 25 | *B. kashiwanohense* TPY25-1 | NDA | 60 | 4 |
| 26 | *B. bifidum* BRS26-2 | NDA | 58 | 6 |
| 26 | *B. longum* TPY26-1 | NDA | 51 | 18 |
| 27 | *B. breve* TPY27 | NDA | 77 | 2 |
| 27 | *B. bifidum* BRS27-3 | NDA | 77 | 4 |
| 28 | *B. bifidum* PV28-2a | NDA | 70 | 5 |
| 28 | *B. bifidum* BSM28-1 | NDA | 57 | 10 |
| 28 | *B. bifidum* BSMd28-2 | NDA | 10 | 1 |
